# Clinical Evidence on the Health Effects of *Aristotelia chilensis* (Maqui Berry) Supplementation: A Systematic Review of Human Trials

**DOI:** 10.3390/antiox15060654

**Published:** 2026-05-22

**Authors:** Patricio Arce-Johnson, Yohaily Rodríguez-Alvarez, Carolina Gabriela Vallejos Sierra, Jesús L. Romero-Romero, Luisbel González, Alain Manuel Chaple Gil

**Affiliations:** 1 Instituto de Ciencias Aplicadas, Facultad de Ingeniería, Universidad Autónoma de Chile, Santiago 8581151, Chile; 2 Agrijohnson Ltda., Miraflores, Santiago 9630000, Chile; 3 Instituto Politécnico Nacional, Centro Interdisciplinario de Investigación Para el Desarrollo Integral Regional Unidad Sinaloa, Guasave 81101, Mexico; 4 Carrera de Odontología, Facultad de Ciencias de la Salud, Universidad Autónoma de Chile, Santiago 8581151, Chile

**Keywords:** *Aristotelia chilensis*, maqui berry, delphinidin

## Abstract

*Aristotelia chilensis* (maqui berry) is a Chilean native fruit rich in anthocyanins with potential antioxidant, glycemic, cardiometabolic, and ocular benefits, but its clinical efficacy remains unclear. This systematic review synthesized and critically appraised human trials evaluating oral maqui supplementation in adults. Following PRISMA 2020 and a PROSPERO-registered protocol, five databases were searched, and risk of bias and certainty of evidence were assessed using RoB 2/ROBINS-I and GRADE. Twelve clinical trials published between 2014 and 2023 were included. Acute studies consistently showed reduced postprandial glucose and modulation of insulin response, whereas chronic interventions showed modest and inconsistent effects on HbA1c, lipid profile, and other cardiometabolic markers. Favorable changes were also reported for oxidative stress biomarkers and autonomic parameters, although these findings were mainly based on surrogate endpoints. The most consistent evidence was observed in the ocular domain, where maqui supplementation improved tear production, dry eye symptoms, and tear inflammatory markers. The overall certainty of evidence ranged from moderate to very low because of methodological heterogeneity, small sample sizes, and short intervention duration. Maqui berry supplementation shows promise, particularly for acute glycemic control and ocular surface health, but larger long-term randomized trials using standardized formulations are needed before definitive clinical recommendations can be made.

## 1. Introduction

*Aristotelia chilensis* (maqui berry), a native species of southern Chile, has emerged as a fruit of increasing interest in nutritional, biomedical, and translational research because of its high contents of anthocyanins and other polyphenolic compounds. Among these, delphinidin derivatives are considered major contributors to its remarkable antioxidant capacity, which has positioned maqui berry as a promising functional food and nutraceutical candidate. In recent years, growing attention has been directed toward its potential role in the prevention and adjunctive management of chronic non-communicable diseases, particularly those in which oxidative stress, inflammation, and metabolic imbalance are central pathophysiological drivers [1].

This interest is part of a broader scientific context in which naturally derived bioactive molecules are being investigated as complementary strategies to counteract oxidative, inflammatory, metabolic, and vascular alterations associated with hyperglycemia and cardiovascular risk. For example, polyphenolic compounds such as hydroxytyrosol have been shown to protect against high-glucose-induced redox imbalance and calcium dysregulation in human erythrocytes, supporting the concept that dietary antioxidants may help attenuate cellular mechanisms linked to diabetic and cardiovascular complications [2].

The biological relevance of maqui berry is supported by an expanding body of experimental evidence indicating that its bioactive compounds exert potent antioxidant and anti-inflammatory effects. These properties are especially important in the context of chronic disorders, where excessive reactive oxygen species production and sustained low-grade inflammation contribute to disease onset and progression. In this regard, maqui-derived extracts have been investigated for their capacity to attenuate oxidative stress, regulate inflammatory signaling, and modulate pathways associated with tissue protection and metabolic adaptation [3,4,5,6,7]. Such findings provide a compelling mechanistic basis for considering maqui berry as a bioactive intervention with potential clinical relevance.

Beyond its antioxidant and anti-inflammatory potential, maqui berry has also been studied for its possible effects on metabolic homeostasis. Available evidence suggests that supplementation with maqui-derived products may influence glucose regulation, lipid metabolism, and related cardiometabolic risk factors, possibly through the inhibition of intestinal sodium–glucose cotransporters (SGLT-1), delayed glucose absorption, and modulation of postprandial glycemic responses, thereby supporting its potential application in conditions such as obesity, metabolic syndrome, and impaired glucose tolerance [1,8,9]. These effects may also extend to cardiovascular protection, given the close relationship between redox balance, dyslipidemia, inflammation, and vascular dysfunction. In addition, emerging evidence points to possible immunomodulatory properties, further broadening the biological and clinical interest surrounding this berry [6,10].

Although several narrative and mechanistic reviews have addressed the phytochemical composition, antioxidant capacity, bioavailability, and physiological effects of maqui berry and anthocyanin-rich foods [1,11,12], the available evidence synthesis has largely remained focused on preclinical mechanisms, metabolic homeostasis, food applications, or broad nutraceutical perspectives. In addition, systematic reviews and meta-analyses have evaluated anthocyanin-rich foods and berries in relation to cardiometabolic risk factors [13,14], but these analyses were not specific to Aristotelia chilensis and did not critically appraise maqui supplementation trials across multiple clinical outcome domains. To date, there has been no systematic review specifically dedicated to critically appraising human clinical trials of Aristotelia chilensis supplementation across multiple health-related outcome domains. This creates an important gap between the growing mechanistic rationale for maqui-derived bioactives and the level of clinical evidence required to support evidence-based nutritional or therapeutic recommendations.

Despite this promising preclinical and translational background, the available human evidence remains limited, heterogeneous, and difficult to interpret in an integrated manner. Several important gaps persist, including the lack of robust data on long-term efficacy and safety, the insufficient characterization of the molecular and biochemical mechanisms underlying the observed effects in humans, and the limited understanding of the bioavailability, metabolism, and pharmacokinetics of its active compounds [1,3,15]. Moreover, comparative clinical studies against other antioxidant-rich foods or polyphenol-based interventions remain scarce, which hampers the contextualization of maqui berry within the broader field of evidence-based functional nutrition [16].

Although individual clinical studies have reported favorable effects of maqui supplementation on postprandial glycemia, oxidative stress biomarkers, cardiometabolic parameters, and ocular surface health, these findings are dispersed across different populations, formulations, doses, and outcome domains. Consequently, the current evidence base lacks a unified and critical synthesis capable of clarifying the consistency, strength, and clinical relevance of these reported effects. In this context, a systematic evaluation of the human literature is warranted. Therefore, the present systematic review aimed to critically appraise and synthesize the available clinical evidence on the effects of standardized maqui berry (*Aristotelia chilensis*) extracts and related formulations on glycemic regulation, oxidative stress biomarkers, cardiometabolic risk factors, and ocular surface outcomes in adult populations.

## 2. Materials and Methods

### 2.1. Study Design and Registration

This systematic review was conducted and reported in accordance with the Preferred Reporting Items for Systematic Reviews and Meta-Analyses (PRISMA) 2020 statement [17]. The review protocol was registered a priori in the International Prospective Register of Systematic Reviews (PROSPERO) under registration number CRD420261362605, before the literature search was initiated. The aim of the review was to identify, critically appraise, and synthesize the available evidence from human clinical trials evaluating the health effects of *Aristotelia chilensis* (maqui berry) supplementation. The eligibility criteria, search strategy, and data extraction framework were predefined and documented in the registered protocol prior to study selection.

### 2.2. Eligibility Criteria

Study eligibility was established according to the Population, Intervention, Comparator, Outcome, and Study design (PICOS) framework. The population of interest comprised adult human participants aged 18 years or older, regardless of baseline health status. The intervention was defined as oral supplementation with maqui berry in any formulation, including standardized extracts, freeze-dried powders, nutraceutical capsules, or polyphenol-rich beverages, administered at any dose and for any duration. No minimum intervention duration was predefined as an eligibility criterion, as established a priori in the PROSPERO-registered protocol, in order to capture both acute mechanistic responses and longer-term physiological adaptations associated with maqui supplementation. Acceptable comparators included placebo, no intervention, an alternative active comparator within a controlled design, or a self-controlled before-and-after scheme with an internal zero-dose control visit. Eligible outcomes included any clinical, biochemical, or patient-reported health endpoint. Eligible study designs were human clinical trials, including randomized controlled trials (parallel-group or crossover), as well as open-label and pilot clinical studies, provided that they reported original data on at least one health-related clinical outcome.

Records were excluded if they corresponded to in vitro or animal studies without a human clinical component; pharmacokinetic or bioavailability studies, including single-dose absorption studies, that reported only absorption, plasma or urinary metabolite concentrations, or compound distribution profiles without assessing any physiological, biochemical, symptom-related, functional, or health-related intervention outcome; narrative reviews, editorials, or opinion papers lacking original clinical trial data; or secondary re-analyses of previously published datasets that did not provide new primary clinical outcomes.

### 2.3. Information Sources and Search Strategy

A systematic literature search was conducted across multiple electronic databases, including PubMed, Scopus, Embase, Web of Science, and the Cochrane Library. The search strategy combined Medical Subject Headings (MeSH) and free-text terms related to the intervention (“*Aristotelia chilensis*”, “maqui”, “maqui berry”, “Delphinol”, “MaquiBright”, “delphinidin”) with terms related to clinical study design (“clinical trial”, “randomized”, “placebo”, “intervention”, “supplementation”). Boolean operators (AND, OR) were used to combine the search terms. No restrictions were applied regarding publication date or language. In addition, the reference lists of all included studies and relevant review articles were manually screened to identify any additional eligible records not retrieved through the electronic search. The full search strategies used for each database are provided in Table A1.

### 2.4. Study Selection

All retrieved records were imported into reference management software, and duplicates were removed. Titles and abstracts were screened independently by two reviewers according to the predefined eligibility criteria. Full-text versions of potentially relevant articles were then obtained and assessed for final inclusion. Any disagreements between reviewers were resolved through discussion and consensus or, when necessary, by consultation with a third reviewer. The study selection process was documented using a PRISMA flow diagram.

### 2.5. Data Extraction

A standardized and pre-piloted data extraction form was used, and data were extracted independently by two reviewers. For each included study, the following information was collected: first author and year of publication, country of origin, study design, type and formulation of the maqui-based product, dose and regimen, target population and main inclusion criteria, nature of the comparator, intervention duration, number of participants randomized and included in the primary analysis, mean age or age range, proportion of female participants, main clinical condition under investigation, organ system involved, clinical specialty, outcome category, key outcomes and measurement instruments, type of outcome (biochemical markers, clinical signs, or patient-reported measures), principal assessment time points, the suitability of the data for quantitative synthesis, main findings and conclusions, and relevant methodological observations. All extracted data were compiled in a structured spreadsheet to ensure transparent data management and to facilitate subsequent subgroup analyses.

### 2.6. Classification and Grouping of Studies

To enable a clinically coherent synthesis, the included clinical trials were organized according to two complementary classification schemes. The first grouped studies by the primary organ system targeted by the intervention, while the second classified them by clinical specialty. This dual taxonomy allowed the evidence to be appraised from both pathophysiological and clinical practice perspectives. When a single trial reported outcomes spanning more than one organ system or clinical discipline, it was assigned to all relevant categories.

### 2.7. Risk of Bias Assessment

The methodological quality of the included studies was assessed independently by two reviewers using design-specific risk-of-bias tools. Randomized controlled trials were evaluated using the Cochrane Risk of Bias tool, version 2 (RoB 2) [18], which assesses five domains: bias arising from the randomization process, bias due to deviations from intended interventions, bias due to missing outcome data, bias in outcome measurement, and bias in selection of the reported result. Each domain was judged as low risk, some concerns, or high risk of bias, and an overall risk-of-bias judgement was assigned to each trial.

Non-randomized studies were assessed using the Risk Of Bias In Non-randomized Studies of Interventions (ROBINS-I) tool [19], which evaluates seven domains: bias due to confounding, bias in participant selection, bias in classification of interventions, bias due to deviations from intended interventions, bias due to missing data, bias in outcome measurement, and bias in selection of the reported result. Each domain was rated as low, moderate, serious, or critical risk of bias, leading to an overall judgement for each study.

Any discrepancies between reviewers were resolved through discussion or consultation with a third reviewer. In addition, methodological concerns identified during data extraction, such as small sample size, lack of dietary standardization, predominance of graphical rather than tabular data presentation, or reliance on non-parametric outcome distributions, were documented and considered in the interpretation of findings.

### 2.8. Data Synthesis

Given the considerable clinical and methodological heterogeneity across the included trials, the feasibility of meta-analytic pooling was evaluated a priori. Substantial heterogeneity was anticipated due to variation in study design (RCTs, crossover trials, open-label studies), intervention formulations (standardized extracts, freeze-dried powders, polyphenol-rich beverages), dosing regimens (30–1000 mg/day), intervention durations (single dose to 90 days), and outcome measures and assessment instruments. In addition, important differences were observed in participant characteristics, including healthy adults, individuals with prediabetes, overweight adults, smokers, and participants with dry eye symptoms, which further limited clinical comparability across studies. For the two outcome categories with the largest number of available data points—postprandial glycemic response (five eligible trials) and ocular surface outcomes (three eligible trials)—a preliminary assessment of statistical heterogeneity was performed by examining the direction and variance of effect estimates across studies. However, formal statistical heterogeneity estimates, such as I^2^, Tau^2^, or chi-square tests, could not be reliably calculated because the studies differed substantially in comparator groups, outcome definitions, reporting formats, time points, and effect measures. In several cases, outcomes were reported graphically, narratively, or using non-equivalent metrics, preventing extraction of a common effect size suitable for pooling. This assessment confirmed that the diversity of interventions and measurement methods precluded meaningful statistical pooling. Therefore, the decision not to perform a meta-analysis was based not only on statistical considerations, but also on formal clinical and methodological heterogeneity, in line with Cochrane Handbook guidance. Therefore, no meta-analysis was performed, and a narrative synthesis was adopted as the primary method of evidence integration, in accordance with the guidance of the Cochrane Handbook for Systematic Reviews of Interventions [20].

## 3. Results

### 3.1. Study Selection

**Figure 1:**
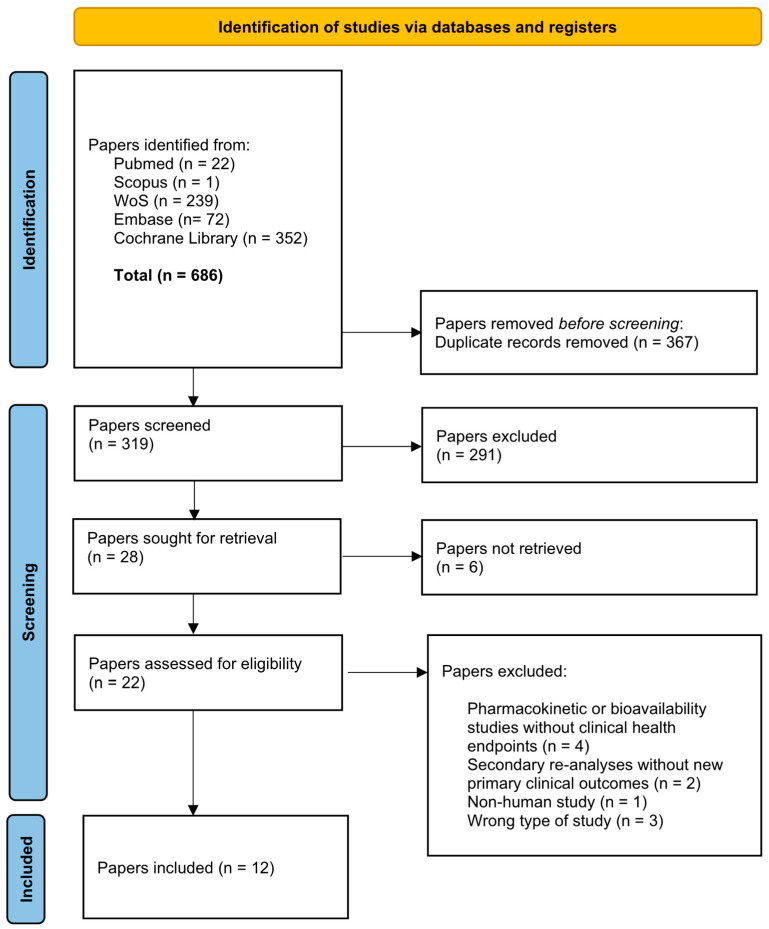
PRISMA flow diagram.

A total of 686 records were identified through systematic searches of five electronic databases: PubMed (*n* = 22), Scopus (*n* = 1), Web of Science (*n* = 239), Embase (*n* = 72), and the Cochrane Library (*n* = 352). After the removal of 367 duplicate records, 319 unique records remained for title and abstract screening. Of these, 291 were excluded for not meeting the predefined eligibility criteria, leaving 28 records for full-text retrieval. Six records could not be retrieved, and 22 articles were therefore assessed for eligibility in full text. Of these, ten were excluded for the following reasons: four were pharmacokinetic or bioavailability studies reporting only urinary or plasma metabolite concentrations without any clinical health endpoint [10,21,22,23]; two were secondary re-analyses of previously published intervention datasets that did not generate new primary clinical outcomes [24,25]; one was an in vitro cell culture study without a human clinical component [5]; three were ineligible study types that did not report original clinical trial data [26,27,28]. Accordingly, 12 studies [29,30,31,32,33,34,35,36,37,38,39,40] were included in the final qualitative synthesis. The complete study selection process is shown in the PRISMA 2020 flow diagram (Figure 1).

### 3.2. Risk of Bias Assessment of Included Studies

**Figure 2:**
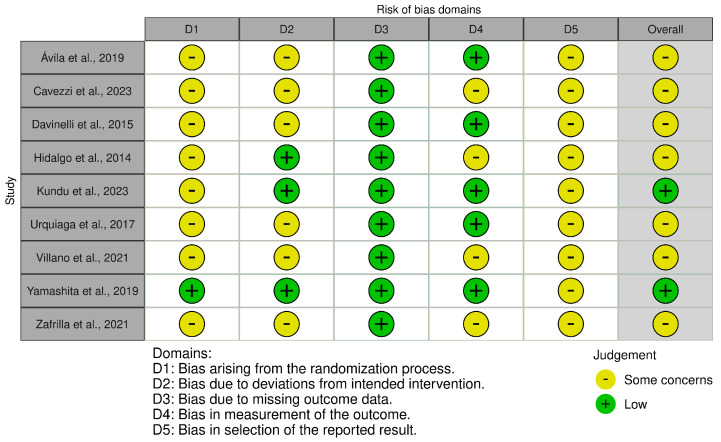
Risk of bias assessment of included studies using the RoB 2 tool [29,30,31,33,35,36,38,39,40].

The methodological quality of the randomized controlled trials, assessed using the RoB 2 tool, is summarized in Figure 2. Overall, most studies were judged as presenting some concerns rather than a low risk of bias. The main sources of concern arose from the randomization process (D1) and deviations from intended interventions (D2), for which all or nearly all studies were rated as having some concerns. By contrast, bias due to missing outcome data (D3) was consistently judged as low risk across all included trials, suggesting adequate handling of incomplete data. Similarly, bias in outcome measurement (D4) was rated as low risk in several studies, although some concerns remained in a minority of trials. Bias in the selection of the reported result (D5) was uniformly judged as raising some concerns. Consequently, only a minority of studies [31,40] achieved an overall low risk of bias, whereas the remainder were classified as having some concerns.

**Figure 3:**
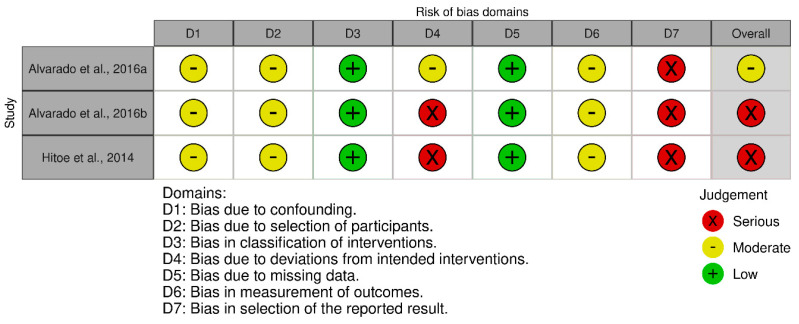
Risk of bias assessment of non-randomized studies using the ROBINS-I tool [32,34,37].

The risk of bias in non-randomized studies, assessed using the ROBINS-I tool, is presented in Figure 3. In contrast to the randomized trials, these studies showed a less favorable methodological profile. All included studies exhibited at least moderate risk of bias across several domains, with particularly important concerns in deviations from intended interventions (D4) and selection of the reported result (D7). Notably, two studies [32,37] were judged to be at serious overall risk of bias, driven primarily by critical issues in these domains. Although bias in classification of interventions (D3) and missing data (D5) was consistently rated as low risk, confounding (D1) and participant selection (D2) remained domains with persistent moderate concerns. Overall, none of the non-randomized studies achieved a low overall risk of bias, underscoring important limitations in internal validity.

### 3.3. Characteristics of Included Studies

The final synthesis included 12 clinical trials published between 2014 and 2023, the main characteristics of which are summarized in Table 1. The predominance of Chilean studies is consistent with the endemic origin of *Aristotelia chilensis* in the temperate rainforests of the southern cone and reflects sustained research interest in this species within its native region.

In terms of study design, the evidence base was methodologically heterogeneous. Four trials used a randomized, double-blind, placebo-controlled, parallel-group design, which is generally considered the reference standard for minimizing bias in supplementation research. One additional trial employed a randomized, triple-blind, three-arm parallel design in which the maqui–citrus beverage matrix was held constant while the sweetener varied across study arms. Two further studies were randomized parallel-group trials in which blinding procedures were not fully reported. Two investigations adopted a randomized crossover design: one acute postprandial study [30] and one mechanistic postprandial study assessing oxidative stress after a high-fat meal [38]. The remaining studies included two open-label, single-arm prospective designs, namely a dose-finding study based on serial oral glucose tolerance tests across four dose levels [34] and a three-month longitudinal intervention [32], together with one open-label pilot study without a placebo comparator [37]. The predominance of open-label and non-placebo-controlled designs among earlier publications suggests a gradual maturation of the field towards more rigorous experimental frameworks over time.

Sample sizes varied substantially, ranging from 10 participants in the smallest acute crossover studies [30,33] to 138 participants in the three-arm parallel Spanish trials [35,39], with a median sample size of approximately 30 participants across the evidence base. Intervention duration was equally variable, ranging from a single acute administration to 90 days of daily supplementation [32], with intermediate regimens of four weeks [36,40], 30 days [29], 60 days [35,39], and 60 days in the ocular pilot study [37]. This wide range of intervention durations precluded direct comparison of effect estimates across studies and highlighted the need to distinguish between acute postprandial responses and chronic physiological adaptations when interpreting the evidence.

The maqui-based products investigated across the included trials covered a broad spectrum of formulations and degrees of standardization. Standardized proprietary extracts were the most frequently used, including Delphinol^®^ (Anklam, Germany), a maqui berry extract standardized for delphinidins and total anthocyanins, which was evaluated in four trials [32,33,34,36], and MaquiBright^®^ (Anklam, Germany), standardized to contain at least 35% total anthocyanins and 25% delphinidins, which was investigated in two ocular health studies [37,40]. Other formulations included a freeze-dried maqui berry powder (Maqui 500^®^, Italy) administered at 1000 mg/day [29], oral nutraceutical capsules containing maqui berry extract [31], maqui–citrus polyphenol-rich beverages sweetened with stevia, sucralose, or sucrose and administered at 330 mL/day [35,39], maqui–lemon beverage blends evaluated for their effects on glycemic response [30], and a Chilean native berry polyphenol concentrate (BPC-350) containing maqui in combination with other berry species [38]. Doses ranged from a single 30 mg capsule of MaquiBright^®^ to 1000 mg/day of freeze-dried powder, although most encapsulated formulations fell within the 60–180 mg/day range. This variation in product type, phytochemical standardization, and dose represented an important source of clinical heterogeneity that must be taken into account when evaluating the consistency and generalizability of the reported effects.

The clinical domains addressed by the included studies clustered into three main areas: endocrine and metabolic outcomes, primarily glycemic regulation, insulin response, and lipid profile, examined in eight trials; ocular surface health, specifically dry eye disease and tear secretion parameters, investigated in three trials; and cardiovascular and oxidative stress outcomes, including autonomic cardiac modulation assessed through heart rate variability indices, addressed in the remaining three trials. Participant profiles ranged from apparently healthy volunteers and healthy adult men to individuals with prediabetes, mild-to-moderate dry eye disease, overweight status, or stress-related autonomic dysregulation, with ages generally spanning 30 to 60 years. Sex distribution was variable and inconsistently reported: two trials enrolled exclusively male participants [30,38], several included predominantly female samples [32,40], and others enrolled mixed-sex cohorts without sex-stratified analyses, thereby limiting the assessment of possible differential responses according to biological sex.

### 3.4. Distribution of Studies by Physiological System

The included trials addressed three principal physiological domains, as summarized in Table 2. The endocrine and metabolic system was the most extensively investigated, encompassing 12 study entries across eight independent trials. Within this domain, the primary focus was glycemic regulation, with five trials evaluating the effects of maqui-derived products on postprandial glucose and insulin responses, either acutely after a standardized meal or glucose load [30,33,34] or following sustained supplementation in individuals with prediabetes [32] or overweight status [39]. Postprandial oxidative stress was examined by Urquiaga et al. [38], who reported attenuation of plasma malondialdehyde and protein carbonyls, together with increased antioxidant capacity, after intake of a Chilean native berry polyphenol concentrate (BPC-350) following a high-fat meal. However, because BPC-350 is a multi-species formulation that includes maqui alongside other Chilean berries, the observed effects cannot be attributed exclusively to *Aristotelia chilensis*, and this limitation was considered in the certainty-of-evidence assessment for the oxidative stress domain. The maqui–citrus beverage studies conducted in overweight Spanish adults [35,39] also contributed to this domain by addressing oxidative, inflammatory, glycemic, and body composition outcomes, although the confounding influence of sweetener type across the intervention arms complicated attribution of the observed effects specifically to maqui. Importantly, these studies did not include a maqui-free placebo arm, and therefore the reported metabolic or inflammatory changes cannot be interpreted as effects attributable exclusively to maqui berry supplementation.

The cardiovascular and autonomic nervous system domain was represented by four study entries across three independent trials. Davinelli et al. [36] showed that four weeks of Delphinol^®^ supplementation reduced plasma oxidized LDL and urinary F2-isoprostanes in overweight adult smokers, although these effects were not sustained at the 40-day post-treatment follow-up and no significant changes were detected in blood pressure, lipid profile, or anthropometric variables. Urquiaga et al. [38] also contributed to this domain through a cardiovascular oxidative stress pathway, reporting reductions in postprandial plasma malondialdehyde and protein carbonyls after berry polyphenol consumption following a high-fat meal. Cavezzi et al. [29] focused specifically on autonomic cardiac function and reported improvements in heart rate variability indices, including SDNN, RMSSD, and total HRV power, along with favorable changes in oxidative biomarkers and mental health-related quality-of-life scores in middle-aged adults with stress-related symptoms after 30 days of supplementation with freeze-dried maqui berry powder.

The ocular and lacrimal surface domain was investigated in three trials. Hitoe et al. [37] provided preliminary pilot evidence that MaquiBright^®^ at 60 mg/day for 60 days increased tear fluid volume, as assessed by the Schirmer test, and improved dry eye symptom scores on the DEQS questionnaire, although the absence of a placebo control limited the interpretability of these findings. Yamashita et al. [40] subsequently confirmed and extended these observations in a randomized, double-blind, placebo-controlled trial, reporting significant improvements in tear production, tear stability, and subjective symptom relief in adults with mild VDT-related dry eye. Kundu et al. [31] further characterized the anti-inflammatory dimension of the ocular response, showing that maqui berry extract supplementation over two months significantly reduced pro-inflammatory tear cytokines, including IL-1β, IL-6, TNF-α, and MMP-9, while increasing the anti-inflammatory mediator IL-10, together with concurrent improvements in OSDI scores and Schirmer test values compared with placebo.

Notably, no clinical trials were identified that directly targeted central nervous system outcomes, such as cognition or mood, or respiratory, musculoskeletal, primary dermatological, systemic immunological, or gynecological endpoints. The current body of human evidence therefore remains concentrated within three interrelated physiological systems, a distribution that partly reflects the established preclinical pharmacology of delphinidins and maqui-derived anthocyanins, and partly the translational and commercial priorities of the research groups working in this field.

### 3.5. Certainty of Evidence Assessment (GRADE)

The certainty of evidence was assessed using the GRADE framework across all included outcomes. Overall, certainty ranged from moderate to very low, and no outcome achieved a high-certainty rating. The most common reasons for downgrading were risk of bias and imprecision, mainly due to methodological limitations in the included studies and wide confidence intervals. Inconsistency was also identified in several comparisons, reflecting variability in effect estimates across studies, whereas indirectness was present in specific outcomes because of heterogeneity in intervention protocols and outcome assessment methods. In addition, surrogate biomarkers and intermediate physiological outcomes were explicitly considered as a source of indirectness in the GRADE assessment. Therefore, outcomes such as HbA1c, HOMA-IR, Ox-LDL, inflammatory biomarkers, antioxidant capacity, and HRV indices were downgraded when they were used as proxies for clinically meaningful endpoints rather than direct measures of disease incidence, cardiovascular events, functional improvement, or long-term clinical benefit. Although surrogate biomarkers such as Ox-LDL, HRV indices, inflammatory cytokines, HbA1c, and antioxidant capacity were considered eligible outcomes within clinical intervention studies, they were distinguished conceptually from purely pharmacokinetic endpoints because they reflect physiological or functional biological responses rather than compound absorption or metabolite distribution alone.

Outcomes supported by a larger number of studies with more consistent findings generally received higher certainty ratings, whereas those based on limited evidence or showing substantial variability were graded as low or very low certainty. A summary of certainty ratings and corresponding effect estimates is presented in Table 3, while the full narrative GRADE assessment, including domain-specific judgements and detailed justifications for downgrading, is provided in Table A2. In the metabolic and cardiometabolic domains, the two reports derived from the same Spanish cohort of 138 overweight adults [35,39] were considered as multiple publications from a single underlying intervention dataset rather than as fully independent trials. Because it was not possible to confirm whether all reported outcomes were prospectively prespecified in a single protocol or derived from subsequent analyses of the same dataset, this overlap was considered when judging risk of reporting bias, multiplicity, imprecision, and certainty of evidence.

## 4. Discussion

### 4.1. Principal Findings

The present systematic review synthesized the available clinical evidence from 12 trials on the effects of *Aristotelia chilensis* supplementation across multiple physiological domains, including glycemic regulation, oxidative stress, cardiovascular function, and ocular surface health. Overall, the evidence suggests that maqui berry-derived products may exert beneficial effects, particularly in acute glycemic modulation, attenuation of oxidative stress, and improvement of dry eye symptoms. However, these findings should be interpreted with caution because of the heterogeneity in study designs, interventions, and outcome measures.

Within the metabolic domain, acute postprandial studies consistently showed reductions in glycemic excursions and the modulation of insulin response following maqui supplementation. Chronic interventions suggested modest improvements in HbA1c and lipid parameters in at-risk populations, although the findings were inconsistent and were often influenced by confounding factors such as formulation matrices and sweetener composition.

With respect to oxidative stress and cardiovascular-related outcomes, the overall direction of effect favored maqui supplementation, with reductions in oxidized LDL and other lipid peroxidation markers, together with improvements in antioxidant capacity and heart rate variability indices. Nevertheless, these outcomes were based predominantly on surrogate biomarkers rather than hard clinical endpoints.

The most consistent evidence was observed in the ocular domain, where maqui supplementation, particularly with standardized extracts such as MaquiBright^®^, demonstrated reproducible improvements in tear production and dry eye symptoms in both pilot and placebo-controlled trials.

### 4.2. Interpretation in the Context of Biological Mechanisms

**Figure 4:**
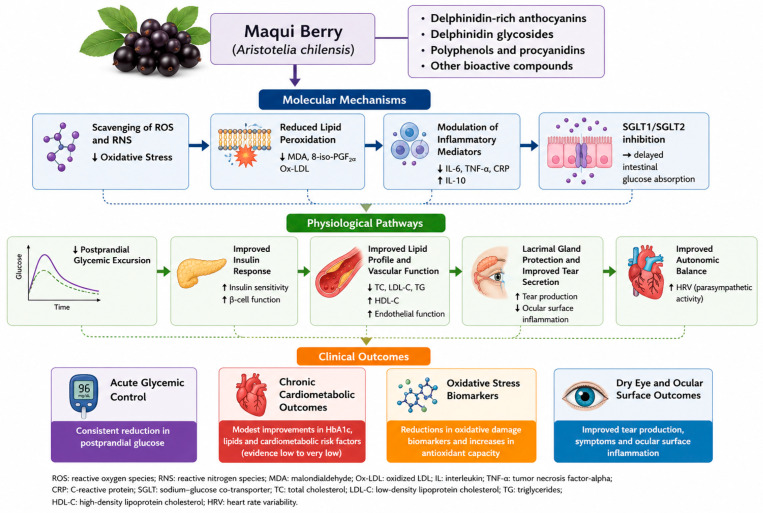
Integrated mechanistic framework of maqui berry bioactives, physiological pathways, and clinical outcomes.

The observed clinical effects are biologically plausible and consistent with the known pharmacological properties of maqui berry, particularly its high contents of anthocyanins and delphinidins. These compounds are recognized for their potent antioxidant capacity, their ability to modulate inflammatory pathways, and their potential effects on glucose transport mechanisms. The interconnected molecular and physiological pathways linking these bioactive compounds to downstream clinical outcomes are summarized in Figure 4.

The acute glycemic improvements reported in several trials may be explained by the inhibition of sodium–glucose cotransporters and delayed intestinal glucose absorption. In addition, the attenuation of postprandial oxidative stress is consistent with the ability of anthocyanins to neutralize reactive oxygen species and reduce lipid peroxidation.

In the ocular domain, the observed improvements in tear production and inflammatory cytokine profiles suggest a dual mechanism involving both the antioxidative protection of the lacrimal glands and modulation of ocular surface inflammation. These mechanisms provide a coherent explanation for the relatively stronger and more consistent findings observed in this domain compared with others.

### 4.3. Comparison with the Previous Literature

The findings of the present systematic review are partially consistent with the broader literature on anthocyanin-rich interventions, although important differences in the magnitude and clinical relevance of the reported effects remain evident.

Regarding cardiovascular outcomes, the present results are in line with those reported by Kimble et al. [41], who identified an inverse association between dietary anthocyanin intake and both coronary heart disease and cardiovascular mortality, suggesting a potential cardioprotective role for these compounds. Similar to the present review, Kimble et al. [41] also reported a lack of consistent associations with other endpoints, such as stroke or total cardiovascular disease, indicating that the effects of anthocyanins may be outcome-specific rather than uniformly distributed across all cardiovascular domains. This pattern is consistent with the heterogeneity observed in the current synthesis, in which cardiometabolic benefits were generally modest and inconsistently reported across trials.

From a metabolic perspective, the present findings are also consistent with the meta-analysis by Rambaran et al. [42], which showed that berry polyphenol supplementation did not produce statistically significant or clinically meaningful reductions in fasting blood glucose or insulin resistance markers in populations at metabolic risk. Specifically, pooled analyses demonstrated negligible effects on fasting blood glucose and no significant improvements in HOMA-IR, despite the inclusion of multiple randomized controlled trials. This agrees with the present review, which identified improvements in glycemic outcomes primarily in acute or postprandial settings rather than in sustained metabolic adaptations.

Similarly, the comprehensive review by Xu et al. [43] supports the notion that anthocyanin-rich berry interventions exert limited effects on traditional cardiometabolic risk markers. Their pooled analyses showed no significant impact on body mass index, blood pressure, or endothelial function in most subgroups, with substantial heterogeneity across studies. These findings reinforce the interpretation that, although anthocyanins have strong biological plausibility as antioxidant and anti-inflammatory agents, their translation into clinically meaningful effects in humans remains inconsistent.

Taken together, these comparisons suggest that the effects observed for *Aristotelia chilensis* are broadly aligned with the wider literature on anthocyanin-rich interventions. The modest and domain-specific benefits identified, particularly in acute glycemic responses and modulation of oxidative stress, appear to reflect a broader pattern observed across berry-derived polyphenols rather than a unique or markedly superior effect attributable exclusively to maqui berry.

### 4.4. Clinical Implications

From a clinical perspective, maqui berry supplementation may represent a complementary strategy for improving specific health parameters, particularly acute postprandial glycemic control in individuals with impaired glucose tolerance, reduction in oxidative stress biomarkers in populations at cardiovascular risk, and management of mild-to-moderate dry eye disease.

However, its clinical applicability remains limited by several factors. First, the magnitude of the observed effects is generally modest. Second, the evidence is derived largely from short-term studies with small sample sizes. Third, heterogeneity in formulations complicates the identification of optimal dosing strategies and limits product standardization.

Therefore, maqui berry should not be regarded as a standalone therapeutic intervention but rather as a potential adjunct within broader preventive or lifestyle-based approaches.

### 4.5. Strengths of the Review

This review contributes to the evidence base in several important respects. First, to the best of our knowledge, it is the first systematic review to comprehensively map the clinical trial evidence on *Aristotelia chilensis* across multiple physiological domains, including metabolic, cardiovascular, and ocular outcomes, thereby providing a cross-domain clinical perspective that domain-specific reviews cannot offer.

Second, the use of both RoB 2 and ROBINS-I, matched to the design of the included studies, ensures that the risk-of-bias assessment is methodologically appropriate across a heterogeneous body of evidence, rather than relying on a single tool for both randomized and non-randomized studies.

Third, the dual classification scheme, based on both physiological system and clinical specialty, enables clinicians and researchers from different disciplines to navigate the evidence according to their area of interest, thereby facilitating translation of the findings into practice and future research.

Fourth, the explicit identification of two publications derived from the same Spanish cohort (*n* = 138), as well as their treatment as multiple reports from a single underlying intervention dataset rather than as fully independent studies, helps prevent the overestimation of the evidence base and provides a more accurate representation of the number of independent clinical datasets available.

### 4.6. Limitations of the Included Evidence

The available body of evidence presents several important limitations that affect the strength and generalizability of the conclusions.

Most studies included small sample sizes, which limits statistical power and increases uncertainty. There was also substantial heterogeneity in study design, including randomized trials, crossover studies, and open-label interventions.

In addition, variability in intervention characteristics, including dose, formulation, and duration, precludes direct comparison across studies. The inclusion of multi-component formulations introduces further confounding and limits causal attribution specifically to maqui. In particular, the maqui–citrus beverage trials combined maqui with different sweeteners in all intervention arms without including a maqui-free placebo comparator, which limited causal attribution of the reported metabolic and inflammatory effects specifically to maqui berry.

Many reported outcomes were surrogate biomarkers rather than clinically meaningful endpoints, thereby restricting inference regarding long-term clinical benefit. These biomarkers, although appropriate for evaluating physiological responses within clinical interventions, should not be interpreted as equivalent to hard clinical outcomes such as disease incidence, hospitalization, or cardiovascular events. This limitation was explicitly incorporated into the GRADE assessment as indirectness, particularly for metabolic, cardiometabolic, oxidative stress, and autonomic outcomes, because most endpoints reflected intermediate biological responses rather than hard clinical outcomes. Moreover, selective reporting and limited protocol transparency emerged as recurring methodological concerns.

A further limitation relates to the inclusion of one trial using a multi-species Chilean berry polyphenol concentrate (BPC-350; Urquiaga et al. [38]), which contains *Aristotelia chilensis* alongside other native berry species. Because the observed effects in this study cannot be attributed exclusively to maqui, its contribution to the oxidative stress evidence domain should be interpreted with particular caution.

Similarly, the maqui–citrus beverage studies by Zafrilla et al. [35] and Villano et al. [39] evaluated complex beverage formulations containing different sweeteners in all intervention arms without including a maqui-free placebo comparator. Therefore, the reported metabolic, inflammatory, and glycemic effects cannot be attributed specifically to maqui berry alone and should be interpreted within the context of the complete beverage matrix and sweetener composition.

Additionally, two publications [35,39] were derived from the same Spanish cohort of overweight adults (*n* = 138) and reported different outcome categories from the same intervention period. Although each publication contributed distinct outcome information, the shared participant pool limits the statistical independence of these contributions within the overall evidence base. Moreover, because the available reports do not clearly establish whether all outcomes were prospectively prespecified in a single protocol or whether some analyses were conducted post hoc, the possibility of multiplicity and selective outcome reporting cannot be excluded. This issue was therefore considered in the risk-of-bias interpretation and contributed to lowering the certainty of evidence for the metabolic and cardiometabolic domains.

### 4.7. Limitations of the Review Process

This review also has limitations inherent to the review process itself. Although a comprehensive search strategy was employed, some relevant studies may not have been identified, particularly unpublished studies or the grey literature.

In addition, six potentially relevant records identified during the screening process could not be retrieved in full text and were therefore excluded from eligibility assessment. Although the number was small relative to the total evidence base, the inability to evaluate these records introduces a potential source of selection bias, as their findings and methodological characteristics could not be considered in the final synthesis.

The use of narrative synthesis, while appropriate given the degree of heterogeneity, limits the ability to quantify effect sizes or formally assess statistical heterogeneity. In addition, the classification of studies across overlapping physiological domains may introduce some degree of interpretative complexity.

### 4.8. Implications for Future Research

Future research should prioritize well-designed, adequately powered randomized controlled trials using standardized maqui formulations and clearly defined dosing regimens.

Long-term studies are needed to evaluate sustained efficacy and safety, as the current evidence is largely limited to short-term interventions. Future trials should also incorporate clinically meaningful endpoints rather than relying predominantly on surrogate biomarkers.

Comparative studies evaluating maqui berry against other polyphenol-rich interventions would be valuable for contextualizing its relative efficacy. Further investigation into pharmacokinetics, bioavailability, and dose–response relationships is also needed to optimize its clinical application.

## 5. Conclusions

This systematic review synthesized the available clinical evidence on the effects of *Aristotelia chilensis* supplementation on glycemic regulation, oxidative stress, cardiometabolic parameters, and ocular surface outcomes in adult populations.

Overall, maqui berry-derived interventions showed a consistent direction of effect towards improved acute postprandial glycemic control and attenuation of oxidative stress biomarkers, together with modest benefits in selected cardiometabolic parameters. The most robust and reproducible clinical effects were observed in the ocular domain, where supplementation improved tear production and reduced dry eye symptoms, as supported by both subjective and objective outcome measures.

The overall certainty of the evidence ranged from moderate to very low, mainly due to methodological limitations, small sample sizes, short intervention periods, and substantial heterogeneity in study design, formulations, and outcome assessment. In particular, the presence of multi-component interventions and the variability in product standardization limit the ability to attribute the observed effects specifically to maqui berry.

Although the current clinical evidence suggests that standardized maqui berry extracts may provide benefits in specific domains, particularly ocular surface health and acute metabolic responses, it remains insufficient to support definitive clinical recommendations. Importantly, the available evidence does not currently demonstrate that maqui berry is superior to other anthocyanin-rich berries or polyphenol-based interventions. Further high-quality, long-term randomized controlled trials using standardized formulations and clinically meaningful endpoints are needed to confirm efficacy, establish optimal dosing strategies, and clarify the therapeutic role of *Aristotelia chilensis* in human health.

## Figures and Tables

**Table 1:** Characteristics of included clinical trials on maqui berry (Aristotelia chilensis) supplementation.

Study	Participants/Condition	Intervention	Design/Duration	Main Findings
Hidalgo et al., 2014 (Chile) [33]	Adults with moderate glucose intolerance/Prediabetes with impaired postprandial glucose	Delphinol, standardized maqui berry extract; single oral dose before a standardized rice meal	Randomized, double-blind, placebo-controlled, crossover/Acute single visit	A single dose of Delphinol reduced postprandial glucose and modified the insulin response versus placebo in prediabetic adults.
Davinelli et al., 2015 (Italy) [36]	Healthy overweight adult smokers/Increased oxidative stress risk	Delphinol, standardized maqui berry extract; daily capsules for 4 weeks	Randomized, double-blind, placebo-controlled, parallel/4 weeks + 40-day follow-up	Delphinol reduced Ox-LDL and urinary 8-iso-PGF2α after 4 weeks, but values returned to baseline after discontinuation; little effect was observed on blood pressure, lipids, or anthropometric variables.
Yamashita et al., 2019 (Japan) [40]	Adults aged 30–60 years with eye fatigue and mild dry eye/visual display terminal (VDT)-related tear hyposecretion	MaquiBright, standardized maqui extract for eye health; 60 mg/day, one capsule before breakfast	Randomized, double-blind, placebo-controlled, parallel/4 weeks	MaquiBright increased tear production, improved tear stability, and reduced subjective dry eye symptoms compared with placebo.
Cavezzi et al., 2023 (Italy) [29]	Apparently healthy adults aged 40–60 years with psycho-physical stress and fatigue/Stress-related autonomic dysregulation	Maqui 500, freeze-dried maqui berry powder; 1000 mg/day (2 × 500 mg capsules)	Randomized, double-blind, placebo-controlled, parallel/30 days	Maqui 500 improved HRV parameters and some oxidative stress biomarkers, suggesting benefits on autonomic balance and stress-related symptoms.
Kundu et al., 2023 (India) [31]	Adults aged 30–50 years with moderate dry eye disease/OSDI >18, TBUT ≤10 and ≥5 s	Maqui berry extract (MBE) capsules; oral supplementation for 2 months	Randomized, double-blind, placebo-controlled, parallel/2 months	MBE significantly improved OSDI scores and Schirmer test results, reduced proinflammatory tear cytokines, and increased IL-10, although TBUT and corneal staining showed no major changes.
Alvarado et al., 2016a (Chile) [34]	Adults with prediabetes/Impaired glucose tolerance or abnormal insulinemia	Delphinol, standardized maqui berry extract; single doses of 0, 60, 120, or 180 mg given 60 min before 75 g OGTT	Open-label, single-arm, dose-finding OGTT study/Four acute visits with washout	Delphinol, particularly at higher doses, reduced postprandial glucose and modulated the insulin response during OGTT in prediabetic adults.
Alvarado et al., 2016b (Chile) [32]	Prediabetic adults managed with lifestyle only/Elevated HbA1c and tendency to dyslipidemia	Delphinol 180 mg, standardized maqui berry extract; 180 mg/day, one capsule in the morning	Open-label, single-arm, prospective/3 months	Delphinol produced a modest but significant reduction in HbA1c and modest improvements in lipid profile; blood pressure changes were small and non-significant, and fasting glucose/insulin showed limited change.
Hitoe et al., 2014 (Japan) [37]	Healthy volunteers with moderately dry eyes/Mild dry eye	MaquiBright, standardized extract (≥35% total anthocyanins, ≥25% delphinidins); 30 or 60 mg/day before breakfast	Open-label pilot/60 days	The 60 mg/day dose significantly increased tear fluid volume and improved dry eye-related quality of life; the 30 mg/day dose showed weaker and less sustained effects. No adverse events were reported.
Ávila et al., 2019 (Chile) [30]	Healthy adult men/Acute postprandial glycemic response	Beverages prepared with maqui extract, lemon juice, or maqui + lemon blends; single dose with a glucose- or rice-based test meal	Randomized crossover, acute postprandial study/Single-meal tests with 120 min follow-up	Maqui and lemon beverages, especially the maqui + lemon blend, significantly reduced postprandial glycemic response and glycemic index of high-GI meals compared with meals alone.
Zafrilla et al., 2021 (Spain) [35]	Overweight adults (*n* = 138)/Metabolic risk and oxidative-inflammatory status	Maqui–citrus beverage (330 mL/day) sweetened with stevia, sucralose, or sucrose	Randomized, parallel RCT/60 days	Sucralose- and sucrose-sweetened beverages increased homocysteine and showed less favorable oxidative/inflammatory responses, whereas stevia increased IL-10 and improved ORAC in subjects with low baseline antioxidant status. These effects should be interpreted as formulation-dependent rather than maqui-specific.
Villano et al., 2021 (Spain) [39]	Overweight adults (*n* = 138)/Risk of impaired glycemic control	Maqui–citrus beverage (330 mL/day) sweetened with stevia, sucralose, or sucrose	Randomized, parallel RCT/60 days	Fasting glucose increased in all groups, but HOMA-IR rose significantly with sucrose and sucralose, not with stevia; stevia showed the relatively least unfavorable glycemic profile. Because no maqui-free placebo was included, these glycemic changes cannot be attributed directly to maqui.
Urquiaga et al., 2017 (Chile) [38]	Healthy male volunteers (*n* = 11)/Postprandial oxidative stress after a high-fat meal	Chilean berry polyphenol concentrate (BPC-350) beverage, including maqui among other berries; beverage alone or incorporated into burger + beverage	Randomized crossover, mechanistic postprandial study/Acute single test days with washout	The berry concentrate reduced postprandial plasma MDA and protein carbonyls and increased antioxidant capacity after a high-fat meal, suggesting attenuation of postprandial oxidative stress.

**Table 2:** Distribution of included studies by physiological system and summary of primary findings with maqui berry supplementation, including positive, null, and unfavorable effects.

Study	System	Positive, Null, and Unfavorable Effects Findings with Maqui
Hidalgo et al., 2014 [33]	Endocrine/metabolic	A single dose of maqui extract reduced postprandial glucose and insulin responses to a rice meal in adults with impaired glucose regulation.
Davinelli et al., 2015 [36]	Cardiovascular/oxidative stress	In overweight adult smokers, 4 weeks of Delphinol reduced plasma oxidized LDL and urinary F2-isoprostanes versus baseline, with minimal changes in BP, lipids and anthropometrics.
Alvarado et al., 2016a [34]	Endocrine/metabolic	In prediabetes, Delphinol acutely reduced postprandial glucose and insulin levels compared with control during oral glucose tolerance testing.
Alvarado et al., 2016b [32]	Endocrine/metabolic/cardiovascular	Three months of daily Delphinol 180 mg in prediabetic adults produced a modest but significant reduction in HbA1c and small favorable changes in LDL, HDL and total cholesterol, with only minor, non-significant effects on blood pressure
Urquiaga et al., 2017 [38]	Endocrine/metabolic	A Chilean berry concentrate, including maqui, attenuated postprandial oxidative stress and increased plasma antioxidant capacity after a high-fat meat meal in healthy men.
Ávila et al., 2019 [30]	Endocrine/metabolic	In healthy men, maqui and maqui + lemon drinks significantly reduced postprandial glycemic response and glycemic index of high-GI glucose and rice meals.
Villano et al., 2021 [39]	Endocrine/metabolic	In overweight adults, 60-day consumption of maqui–citrus beverages with sucrose, sucralose, or stevia was associated with increases in fasting plasma glucose (sucrose: +26%; sucralose: +20%; stevia: +11%) and elevated HOMA-IR with sucrose and sucralose, while stevia showed a comparatively less unfavorable glycemic profile. No maqui-specific effect could be isolated from the sweetener effect.
Zafrilla et al., 2021 [35]	Endocrine/metabolic	In overweight adults, maqui–citrus beverages containing different sweeteners produced modest changes in oxidative and inflammatory markers; however, the absence of a maqui-free placebo prevented attribution of these effects specifically to maqui berry.
Cavezzi et al., 2023 [29]	Endocrine/metabolic	A maqui-based nutraceutical improved HRV parameters, mental SF-12 scores, and prevented adverse changes in body composition compared with controls over 30 days.
Hidalgo et al., 2014 [33]	Cardiovascular/autonomic	Maqui extract acutely enhanced endothelial function, supporting a vascular protective effect.
Davinelli et al., 2015 [36]	Cardiovascular/autonomic	Delphinol reduced oxidative stress biomarkers linked to cardiovascular risk in overweight smokers.
Urquiaga et al., 2017 [38]	Cardiovascular/oxidative stress	Berry concentrate including maqui blunted postprandial oxidative stress after a high-fat meal, suggesting vascular protection.
Cavezzi et al., 2023 [29]	Cardiovascular/autonomic nervous system	Maqui nutraceutical improved autonomic balance (higher SDNN, RMSSD, VLF and total HRV power) compared with no supplementation.
Hitoe et al., 2014 [37]	Ocular/lacrimal/surface	In mild dry eye, an open-label MaquiBright pilot (30 vs. 60 mg/d) increased Schirmer test values and improved symptom scores over 60 days, without a placebo control.
Yamashita et al., 2019 [40]	Ocular/lacrimal/surface	In subjects with eye dryness, MaquiBright increased Schirmer test values and improved symptoms versus placebo.
Kundu et al., 2023 [31]	Ocular/lacrimal/surface	Maqui berry extract increased tear production, reduced OSDI scores and significantly decreased pro-inflammatory tear cytokines while increasing IL-10 compared with placebo.

**Table 3:** Certainty of Evidence Overview according to the GRADE approach. Due to substantial heterogeneity precluding meta-analytic pooling, effect estimates are presented as the direction and magnitude of effects reported narratively across included trials, not as pooled quantitative estimates.

Outcome	Study Design	Studies (Participants)	Risk of Bias	Direction and Narrative Summary of Effect	Certainty (GRADE)	Comments
Acute glycemic control and postprandial metabolic response [30,33,34]	Randomized crossover trials, placebo-controlled acute trials, and open-label exploratory studies	4 studies (99 participants in main analyses)	Moderate to serious	Consistent direction of effect toward reduced postprandial glucose and insulin response across 4 trials; glucose reductions of approximately 15–30% of peak postprandial values across individual studies.	Low	Most acute studies reported lower postprandial glucose, reduced glycemic peak, or a favorable modulation of insulin response after maqui extract or maqui-based beverages. However, studies differed substantially in population, matrix, dose, comparator, and design, and several had small samples or lacked a parallel control group.
Chronic glycemic and cardiometabolic markers with standardized maqui extract [32]	Open-label prospective study	1 study	Moderate to serious	Modest HbA1c reduction and small favorable lipid changes were reported after Delphinol supplementation.	Very low	Evidence was based on a single open-label study with short follow-up and surrogate metabolic outcomes; therefore, causal interpretation remains limited.
Metabolic and inflammatory markers with maqui–citrus beverage formulations [35,39]	Randomized parallel trials derived from the same cohort	2 reports from one cohort	Moderate to serious	Mixed formulation-dependent effects were reported. Fasting glucose and HOMA-IR changes were observed across sweetener groups.	Very low	These studies used maqui–citrus beverages containing different sweeteners and did not include a maqui-free placebo comparator. Therefore, increases in fasting glucose or HOMA-IR cannot be attributed to maqui berry itself, but should be interpreted as formulation-dependent outcomes potentially influenced by sweetener composition.
Ocular surface outcomes, tear production, and dry eye symptoms [31,37,40]	Placebo-controlled randomized trials plus one open-label pilot study	3 studies (107 participants)	Moderate	Consistent direction toward improved Schirmer test values and reduced dry eye symptom scores across 3 trials including 2 RCTs; pro-inflammatory tear cytokines reduced in one placebo-controlled trial.	Moderate	This was the most consistent clinical domain. Across the available studies, maqui supplementation generally increased Schirmer test values and improved dry eye symptoms, although not all ocular parameters improved uniformly and one supporting study lacked placebo control.
Oxidative stress, vascular/autonomic, and antioxidant outcomes [29,36,38]	Randomized placebo-controlled trials and acute crossover mechanistic studies	3 studies (113 participants)	Moderate	Directionally favourable for oxidative biomarkers in 3 of 4 trials; HRV improvements reported in one RCT. Effects not attributable exclusively to maqui in one multi-berry trial.	Low	The overall direction of effect favored maqui for oxidative stress biomarkers, antioxidant capacity, endothelial or autonomic-related outcomes. Nevertheless, the evidence is based mainly on surrogate markers, short follow-up, small samples, and in one study a mixed-berry concentrate that was not attributable to maqui alone.
Safety and tolerability [31,35,37,39,40]	Small clinical trials, crossover studies, and open-label interventions	Multiple studies (>300 cumulative participants across summarized human trials)	Moderate to serious	No serious adverse events reported across included trials; safety was a secondary outcome in all studies, limiting interpretability.	Low	Short-term use was generally well tolerated, and no consistent serious safety signal was identified in the included human studies. Still, safety was usually a secondary outcome, follow-up was short, and the available evidence is underpowered to rule out uncommon or longer-term adverse effects.

## Data Availability

No new data were created or analyzed in this study. Data sharing is not applicable to this article.

## Abbreviations

The following abbreviations are used in this manuscript:

BPC-350	Chilean native berry polyphenol concentrate
BP	Blood pressure
DEQS	Dry Eye-Related Quality-of-Life Score
GI	Glycemic index
GRADE	Grading of Recommendations Assessment, Development and Evaluation
HbA1c	Glycated hemoglobin
HDL	High-density lipoprotein
HOMA-IR	Homeostatic Model Assessment of Insulin Resistance
HRV	Heart rate variability
IL-1β	Interleukin-1 beta
IL-6	Interleukin-6
IL-10	Interleukin-10
LDL	Low-density lipoprotein
MBE	Maqui berry extract
MDA	Malondialdehyde
MeSH	Medical Subject Headings
MMP-9	Matrix metalloproteinase-9
OGTT	Oral glucose tolerance test
ORAC	Oxygen radical absorbance capacity
OSDI	Ocular Surface Disease Index
Ox-LDL	Oxidized low-density lipoprotein
PICOS	Population, Intervention, Comparator, Outcome, and Study design
PRISMA	Preferred Reporting Items for Systematic Reviews and Meta-Analyses
PROSPERO	International Prospective Register of Systematic Reviews
RCT	Randomized controlled trial
RMSSD	Root mean square of successive differences
RoB 2	Risk of Bias 2
ROBINS-I	Risk Of Bias In Non-randomized Studies of Interventions
SDNN	Standard deviation of normal-to-normal intervals
SF-12	12-Item Short Form Health Survey
TBUT	Tear breakup time
TNF-α	Tumor necrosis factor alpha
VDT	Visual display terminal
VLF	Very low frequency

## Appendix A

**Table A1:** Search queries for each database.

Database	Query	Filters
Pubmed	(“*Aristotelia chilensis*” [MeSH Terms] OR “*Aristotelia chilensis*” [Title/Abstract] OR “maqui” [Title/Abstract] OR “Chilean wineberry” [Title/Abstract] OR “maqui berry” [Title/Abstract] OR “*A. chilensis*” [Title/Abstract])	Filters applied: Clinical Study, Clinical Trial, Controlled Clinical Trial, Randomized Controlled Trial.
Embase	‘aristotelia chilensis’/exp OR ‘aristotelia chilensis’:ti,ab,kw OR ‘maqui’:ti,ab,kw OR ‘chilean wineberry’:ti,ab,kw OR ‘maqui berry’:ti,ab,kw OR ‘a. chilensis’:ti,ab,kw	AND (‘article’/it OR ‘clinical trial’/it)
Scopus	TITLE-ABS-KEY “*Aristotelia chilensis*” OR “maqui” OR “Chilean wineberry” OR “maqui berry” OR “*A. chilensis*”)	AND (LIMIT-TO (DOCTYPE, “ar”))
Web of Science	TS = (“*Aristotelia chilensis*” OR “maqui” OR “Chilean wineberry” OR “maqui berry” OR “*A. chilensis*”)	Articles

**Table A2:** Detailed narrative GRADE evidence profile, including domain-level assessments (risk of bias, inconsistency, indirectness, imprecision, and publication bias) and explicit reasons for downgrading for each outcome.

Outcome	Study Characteristics	Main Findings	Certainty of Evidence
Glycaemic control and insulin response [30,32,33,34,35,39]	Acute and short-term nutritional interventions, mainly small RCTs and pre-post studies; about 8 studies and 250–300 adults; risk of bias from some concerns to high.	Short-term benefit predominated. Acute trials in prediabetic and healthy adults generally reduced postprandial glucose excursions and/or glycaemic index, with modest insulin modulation. Chronic studies suggested small HbA1c and lipid improvements, but fasting glucose and insulin were inconsistent. In overweight adults, maqui–citrus beverages with sucrose or sucralose were linked to slight worsening of fasting glycaemia and HOMA-IR, making the maqui-specific effect difficult to isolate.	Low. Downgraded for risk of bias, inconsistency, and indirectness because of open-label single-arm designs, small single-centre samples, heterogeneous interventions, and reliance on surrogate metabolic markers with short follow-up.
Cardiometabolic and vascular risk markers [29,35,38]	Short-term randomized and non-randomized mechanistic trials, including 4–12 week interventions and single-meal tests; about 6–7 studies and 200–250 adults; some concerns to high risk of bias.	Biomarker findings were directionally favourable. Maqui supplementation reduced oxidized LDL, urinary F2-isoprostanes, and postprandial oxidative stress in several studies, while a maqui-based nutraceutical improved heart rate variability and mental quality-of-life scores. However, changes in blood pressure and standard lipid profiles were modest, inconsistent, and often not clinically relevant.	Low. Downgraded for risk of bias, inconsistency, and indirectness due to small trials, unclear or partial blinding, industry funding, varying formulations and co-interventions, and use of surrogate cardiovascular markers rather than hard outcomes.
Ocular surface function and dry eye symptoms [31,37,40]	One well-designed RCT plus small open-label pilot studies; 3 main trials and about 100–110 adults with dry-eye-related complaints; low risk in main RCTs, high in pilot studies.	This was the most consistent domain. Standardized maqui extracts, such as MaquiBright, increased Schirmer test values and improved dry eye symptom scores versus placebo over 4–8 weeks. Supportive evidence also suggested dose-related increases in tear volume and reductions in pro-inflammatory tear cytokines, together with increased IL-10.	Moderate. Downgraded one level for imprecision and indirectness because total sample size was modest and outcomes were based on short-term tear tests and symptom scales rather than longer-term clinical endpoints.
Safety and adverse effects [36,37]	Safety information came from metabolic, cardiovascular, and ocular maqui trials, mostly short-term RCTs and small pre-post studies; combined samples were in the low hundreds; safety was usually a secondary endpoint.	No clear harm was identified. No serious adverse events clearly attributable to maqui were reported, and liver enzymes, renal markers, and routine clinical chemistry generally remained within normal ranges. Mild events such as transient gastrointestinal discomfort or headache were infrequent and similar to controls when placebos were used. Interpretation remains limited by small samples, selected populations, short durations, and lack of long-term pharmacovigilance.	Low. Downgraded for imprecision, indirectness, and risk of bias because exposure time was limited, few events were captured, vulnerable populations were not studied, and safety outcomes were not always systematically collected or adjudicated.
